# A Single Base-Pair Change in 2009 H1N1 Hemagglutinin Increases Human Receptor Affinity and Leads to Efficient Airborne Viral Transmission in Ferrets

**DOI:** 10.1371/journal.pone.0017616

**Published:** 2011-03-02

**Authors:** Akila Jayaraman, Claudia Pappas, Rahul Raman, Jessica A. Belser, Karthik Viswanathan, Zachary Shriver, Terrence M. Tumpey, Ram Sasisekharan

**Affiliations:** 1 Harvard-MIT Division of Health Sciences and Technology, Singapore-MIT Alliance for Research and Technology, Department of Biological Engineering, Koch Institute for Integrative Cancer Research, Massachusetts Institute of Technology (MIT), Cambridge, Massachusetts, United States of America; 2 Influenza Division, Centers for Disease Control and Prevention (CDC), Atlanta, Georgia, United States of America; Virginia Polytechnic Institute and State University, United States of America

## Abstract

The 2009 H1N1 influenza A virus continues to circulate among the human population as the predominant H1N1 subtype. Epidemiological studies and airborne transmission studies using the ferret model have shown that the transmission efficiency of 2009 H1N1 viruses is lower than that of previous seasonal strains and the 1918 pandemic H1N1 strain. We recently correlated this reduced transmission efficiency to the lower binding affinity of the 2009 H1N1 hemagglutinin (HA) to α2→6 sialylated glycan receptors (human receptors). Here we report that a single point mutation (Ile219→Lys; a base pair change) in the glycan receptor-binding site (RBS) of a representative 2009 H1N1 influenza A virus, A/California/04/09 or *CA04/09*, quantitatively increases its human receptor-binding affinity. The increased human receptor-affinity is in the same range as that of the HA from highly transmissible seasonal and 1918 pandemic H1N1 viruses. Moreover, a 2009 H1N1 virus carrying this mutation in the RBS (generated using reverse genetics) transmits efficiently in ferrets by respiratory droplets thereby reestablishing our previously observed correlation between human receptor-binding affinity and transmission efficiency. These findings are significant in the context of monitoring the evolution of the currently circulating 2009 H1N1 viruses.

## Introduction

On June 11, 2009, the World Health Organization raised the global pandemic alert to phase 6, the pandemic phase [1], in response to the emergence and global spread of a novel H1N1 influenza A virus (referred to henceforth as 2009 H1N1). Phylogenetic analysis of the 2009 H1N1 virus indicated that it emerged from reassortment of recent North American H3N2 and H1N2 swine viruses (avian/human/swine ‘triple’ reassortant viruses) with Eurasian avian-like swine viruses [2], [3], [4]. Owing to a decline in the number of cases of 2009 H1N1 viral infection in 2010, the WHO declared the end of the 2009 H1N1 pandemic in early August 2010. At this time, there were more than 600,000 laboratory confirmed cases of 2009 H1N1 infection and over 18,449 deaths as reported by the WHO [5]. Currently the 2009 H1N1 virus circulates as a seasonal strain causing dispersed infection worldwide. A representative 2009 H1N1 strain, A/California/04/09 (*CA04/09*), was chosen for trivalent seasonal flu vaccine in 2010. Epidemiological [6], [7], [8] and transmission studies using the ferret (*Mustela putorius furo*) animal model [9] have shown that the efficiency of transmission of these viruses is lower than that of the prototypic 1918 pandemic H1N1 (A/South Carolina/1/1918 or *SC18*) and the 2007-08 seasonal H1N1 (A/Brisbane/59/2007 or *Bris07*) influenza A viruses. Moreover the transmission of 2009 H1N1 virus via respiratory droplets was delayed after exposure as compared to the *Bris07* virus [9].

Using prototypic pandemic strains, such as 1918 H1N1 and 1957-58 H2N2, we have previously demonstrated that the binding affinity of HA to human receptors is one important factor that correlates with the transmissibility of the virus via respiratory droplets in ferrets [10], [11], [12]. More recently, we extended this finding to 2009 H1N1 by characterizing the glycan-receptor binding specificity and affinity of the *CA04/09* HA. These studies demonstrated that *CA04/09* HA bound specifically to human receptors on a glycan array platform and on human upper respiratory epithelia, and showed no detectable binding to α2→3 sialylated glycans (referred to as avian receptors) [9]. This observed human receptor specificity of 2009 H1N1 HA was consistent with similar studies by others [13]. The human receptor-binding affinity of *CA04/09* HA, however, was substantially lower than that of SC18 HA [9]. The lower human receptor-binding affinity of *CA04/09* HA correlated with the observed lower efficiency of airborne transmission of the *CA04/09* virus.

In the present study, using a combination of homology-based structural modeling of HA RBS and its interactions with glycan receptor and HA sequence analysis we sought to identify mutations in the RBS that would increase human receptor binding affinity of *CA04/09* HA. We demonstrate that a single Ile219→Lys mutation in the RBS of 2009 H1N1 HA (*CA04/09mut1*) substantially increases its human receptor affinity in comparison with that of *CA04/09* HA. We tested the effect of this mutation experimentally by site-directed mutagenesis and recombinant expression of mutant HA and analyzed this mutant HA using a dose-dependent glycan array assay to quantify HA-glycan binding affinity. Finally, we constructed a *CA04/09* virus harboring the Ile219→Lys HA mutation using reverse genetics and showed that this mutant virus transmits efficiently via respiratory droplets in ferrets. Our findings reemphasize the previously established correlation between human receptor-binding affinity and transmission efficiency. Furthermore, our approach to identify HA mutations that improve human receptor affinity and transmissibility of the virus is significant in the context of surveillance of the evolution of the currently circulating 2009 H1N1 virus.

## Results

### Rationale for designing Ile219→Lys mutation on *CA04/09* HA

The RBS of *CA04/09* HA contains the hallmark amino acids Asp190 and Asp225 that are characteristic of all the human-adapted H1N1 HAs [10], [14], [15], [16]. Our previous comparison of amino acids in the RBS of *CA04/09* and *SC18* HA revealed differences at positions 145, 186, 189, 219, and 227 [17]. To understand the effect of the amino acid differences on the molecular interactions in the RBS, we constructed a structural model of *CA04/09* HA with a human receptor using the recently solved crystal structure of this HA [18]. Analysis of this model indicates that Lys145 in *CA04/09* HA provides an additional anchoring contact for the sialic acid. The residues at 186, 187, and 189 are positioned to form an interaction network with Asp190. The residues 219 and 227 in turn influence the positioning of residue 186.

**Figure 1:**
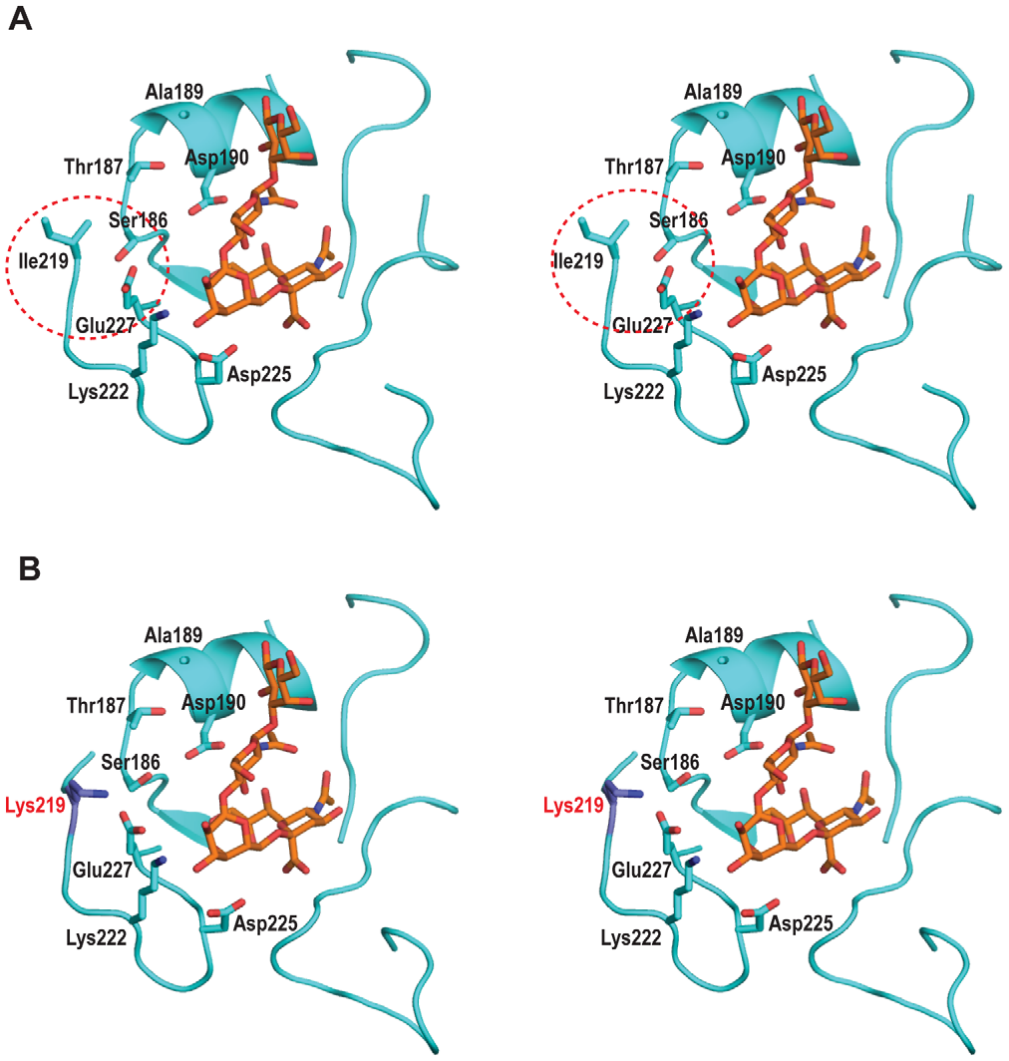
Fixing the mismatched interaction in RBS of *CA04/09* HA. **A**, Structural complex of RBS of *CA04/09* HA with human receptor (mismatched Ile219 - Glu227 interaction highlighted in *red* circle). **B**, RBS of *CA04/09mut1* HA in complex with human receptor where Lys219 (highlighted in *red*) makes ionic contacts with Glu227. The structural complexes are shown in stereo with RBS represented as a cartoon schematic with side chains of key amino acids. The substituted amino acids are labeled *red*. Stick representation of human receptor is shown with carbon atoms in *orange*.

Comparison of residues 219 and 227 across a number of human-adapted HA sequences reveals that either both amino acids are hydrophobic, such as Ala219 and Ala227 as observed in *SC18* HA, or they are charged residues, such as Lys219 and Glu227 as observed in *Bris07* HA (**Figure S1**). In contrast, the 2009 H1N1 influenza A virus HAs have a combination of Ile219 and Glu227 that are highly conserved and that result in a set of interactions that are neither fully hydrophobic nor fully charged (**Figure 1A**). This mismatched combination of residues potentially disrupts the positioning of residues 186, 189 and 190 that form a stable network of inter-residue interactions. This network also plays an important role in positioning Asp190 for optimal contact with α2→6 glycans. To correct the mismatched combination of residues in the RBS of *CA04/09* HA, our strategy was to generate a single mutant; Ile219→Lys (*CA04/09mut1*), that would generate a stable ionic interaction between Lys219 and Glu227. To confirm this analysis, we constructed a homology-based structural of *CA04/09mut1* HA in complex with human receptor. Based on this model we observed that Lys219 and Glu227 are positioned to make ionic contacts that would facilitate the stabilization of interaction network involving residues 186, 187, 189 and 190 (**Figure 1B**). We note that the mismatched combination can also be fixed by making this interaction network hydrophobic. However this would involve at least two amino acid substitutions, Ser186→Pro and Glu227→Ala, to make this interaction network similar to that observed in *SC18* HA (**Figure S1**).

### Quantifying human receptor-binding affinity of *CA04/09mut1* HA

A dose-dependent binding of HA to representative human and avian receptors on a biotin-streptavidin based glycan array platform permitted quantification of binding affinity using an apparent binding constant *K_d_'* [10]. The parameter *K_d_'* was calculated by fitting the binding data (over a range of HA concentrations) using the Hill equation for multivalent binding [10]. Given that HA is a homotrimer, a trimeric HA unit comprises of three identical HA monomers (with one RBS per monomer). The spatial arrangement of the biotinylated glycans in the wells of the streptavidin plate array favors binding to only one of the three HA monomers in the trimeric HA unit. Therefore in order to specifically enhance the multivalency in the HA-glycan interactions, the recombinant HA was pre-complexed with the primary and secondary antibodies in the ratio of 4∶2∶1 (HA: primary: secondary) as described previously [10], [19]. The identical spatial arrangement of 4 trimeric HA units in the pre-complex relative to the glycans on the array platform, homogenizes the avidity effects across different HAs and hence permits quantitative comparison between glycan binding affinities of different HAs.

**Figure 2:**
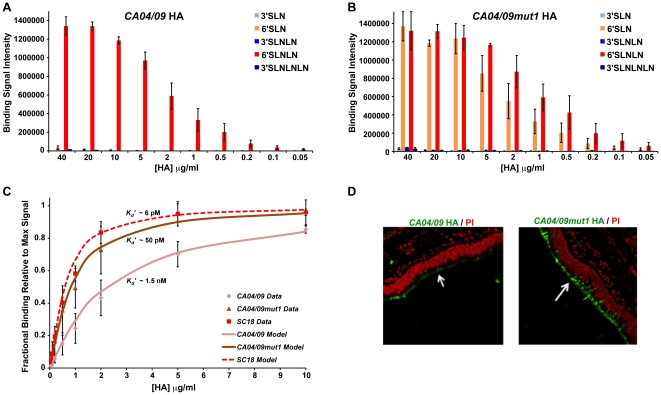
Glycan receptor-binding property of *CA04/09* and *CA04/09mut1* HAs. Dose dependent direct glycan array binding of **A**, *CA04/09* and **B**, *CA04/09mut1* HAs. The mutant HA shows broader α2→6 sialylated glycan receptor specificity (shows binding to both 6′SLN and 6′SLN-LN) compared to *CA04/09* HA. Both HAs show minimal binding to α2→3 sialylated glycans. **C**, 6′SLN-LN binding curves of *CA04/09*, *CA04/09mut1* and *SC18* HAs. The experimental data (disconnected markers indicated using “Data”) is shown along with the theoretical binding curve (line indicated using “Model”) generated as described in *Methods* section. The calculated binding constant *K_d_*' values are shown on top of the binding curves. **D**, Staining of human tracheal tissue sections with *CA04/09* and *CA04/09mut1* (*green*) against propidium iodide (*red*). The apical surface of the tracheal tissue sections is indicated using a *white arrow*.

Using this approach, we performed a dose-dependent glycan array analysis of *CA04/09* and *CA04/09mut1* HAs (**Figure 2A–B**). In comparison with *CA04/09* HA, which showed specific binding to only 6′SLN-LN, *CA04/09mut1* HA showed binding to both the representative human receptors (6′SLN and 6′SLN-LN) on the array. No detectable binding to avian receptors was observed with both *CA04/09* and *CA04/09mut1* HAs. To compare the binding affinity of *CA04/09* and *CA04/09mut1* HAs to a representative human receptor, we calculated the binding constant *K_d_'* from the 6′SLN-LN binding curves (**Figure 2C**). The 6′SLN-LN binding affinity of *CA04/09mut1* HA (*K_d_'*∼50 pM) is about 30-fold higher than that of *CA04/09* HA (*K_d_'*∼1.5 nM). The human receptor-binding affinity of *CA04/09mut1* HA quantified using *K_d_'* is in the same range (picomolar) as that of *SC18* HA (*K_d_'*∼6 pM). Therefore fixing the mismatched combination of residues at 219 and 227 in the RBS of 2009 H1N1 HA substantially increases its human receptor-binding affinity. We also verified that the dose-dependent binding profile of recombinant *CA04/09mut1* HA was consistent with that of the whole virus harboring the Ile219→Lys HA mutation on our glycan array platform (**Figure S2**).

### Binding of *CA04/09mut1* HA to human tracheal tissue sections

Human upper respiratory tissues are the primary targets for infection by human-adapted influenza A viruses for efficient viral transmission by respiratory droplets [10], [20]. The tracheal tissue section, a representative human upper respiratory tissue, has been shown to predominantly express α2→6 sialylated glycan receptors in the ciliated and non-ciliated (goblet) cells in the apical surface of the pseudostratified epithelium [20], [21], [22], [23], [24]. In contrast, the human alveolus, a representative deep lung tissue, has been shown to predominantly express α2→3 sialylated glycan receptors [20], [22], [25]. The binding of *CA04/09* and *CA04/09mut1* HAs to physiological glycan receptors was assessed by staining human tracheal and alveolar tissue sections with these HAs. Consistent with their minimal α2→3 glycan receptor-binding on the array, no visible alveolar staining was observed with these HAs (data not shown). On the other hand, both *CA04/09* and *CA04/09mut1* HAs stained the apical surface of the human tracheal epithelium (**Figure 2D**) consistent with their human receptor-binding on the array. Furthermore, *CA04/09mut1* HA showed more intense tracheal staining in comparison with *CA04/09* HA, which correlated with the broader human receptor specificity [binding to both 6′SLN and 6′SLN-LN] and increased human receptor affinity of the mutant HA on the glycan array. The sialic acid specific binding of HAs to the tracheal tissue sections was confirmed by loss of staining upon pre-treatment of the tissue sections with Sialidase A (from *Arthrobacter ureafaciens*), an enzyme that cleaves terminal sialic acid from both avian and human receptors (data not shown).

### Transmissibility of a reverse genetics wild-type and mutant CA04/09 virus in ferrets

For respiratory droplet transmission experiments, three ferrets were inoculated intranasally (i.n.) with 10^6^ PFU (plaque forming units) of virus. Approximately 24 hours later, inoculated-contact animal pairs were established by placing a naïve ferret in each of three adjacent cages with perforated sidewalls, allowing exchange of respiratory droplets without direct or indirect contact [9]. Inoculated and contact animals were monitored for clinical signs over a 16 day period and transmission was assessed by titration of infectious virus in nasal washes and confirmed by the detection of virus specific antibodies in convalescent sera.

**Figure 3:**
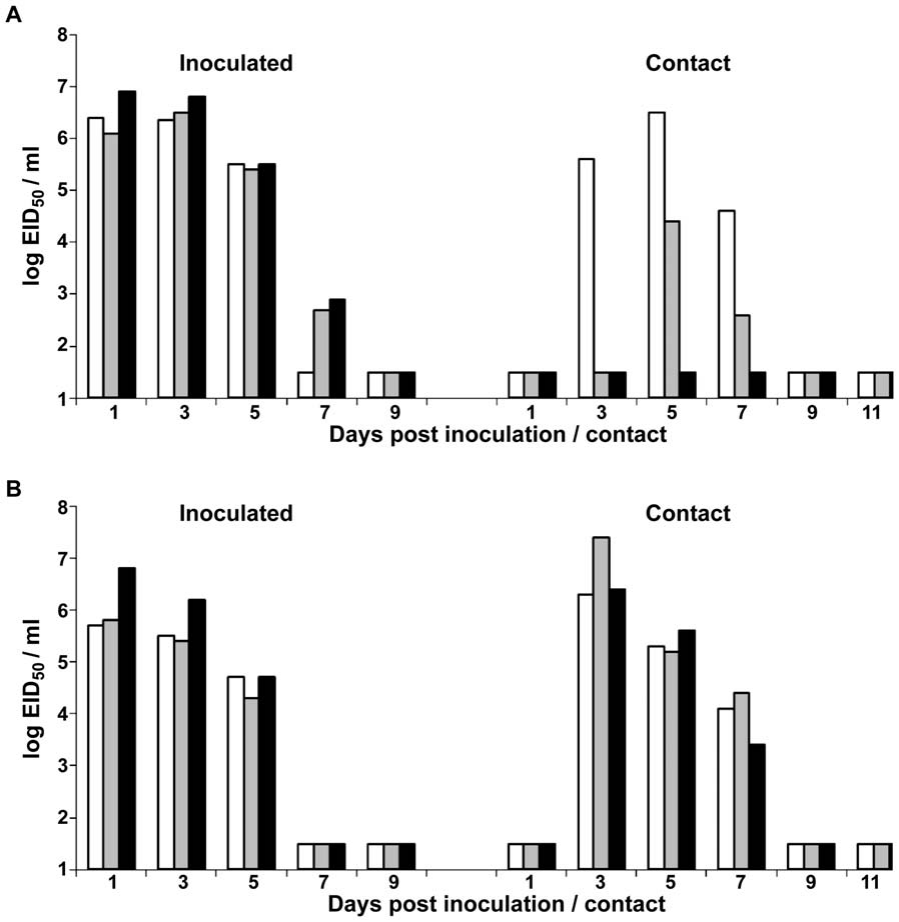
Respiratory droplet transmissibility of *rgCA04/09* and *rgCA04/09 HA Ile219Lys* mutant virus. Three ferrets were intranasally inoculated with 10^6^ PFU of *rgCA04/09* (**A**) or *rgCA04/09 HA Ile219Lys* mutant virus (**B**). A naïve ferret was placed in an adjoining cage to each inoculated ferret and viral shedding in nasal washes was assessed every other day for the inoculated (left) and contact (right) ferrets. Results from individual ferrets are presented. Limit of detection is 10 PFU/mL.

Ferrets inoculated with the *CA04/09* (*rgCA04/09*) virus, created by reverse genetics, showed no substantial clinical signs and only displayed transient weight loss during the first week of infection (**Table 1**). Ferrets inoculated with *rgCA04/09* virus shed high peak mean titers of infectious virus in nasal washes as early as day 1 p.i. (10^6.1–6.9^ PFU/ml), that were sustained at titers of ≥10^2.9^ PFU/ml for 7 days p.i. The *rgCA04/09* virus shedding showed similar kinetics to mutant virus harboring the Ile219→Lys amino acid mutation in HA, which was sustained for 5 days in ferrets at titers of ≥10^4.3^ PFU/ml. Consistent with the experimental transmission data obtained with wild type *CA04/09* H1N1 virus [9], *rgCA04/09* virus did not spread by respiratory droplet to every contact ferret and transmission was delayed by five days post exposure in one of two contact ferrets (**Figure 3**). Seroconversion confirmed the virus shedding data of 2/3 transmission events (**Table 1**). The reduced respiratory droplet transmission suggests that additional virus adaptation in mammals may be required to reach the high-transmissible phenotypes observed with seasonal H1N1 or the 1918 pandemic virus [9], [14]. Mutation of the HA at amino acid position 219 (Ile219->Lys) resulted in a virus (*rgCA04/09 HA Ile219Lys*) that efficiently transmitted via respiratory droplets to all of the contact ferrets which shed virus by day 3 post-contact (p.c.) (**Figure 3**). Despite the differences in transmissibility of the parental *rgCA04/09* virus and the *rgCA04/09 HA Ile219Lys* mutant virus, similar virus replication kinetics, and comparable levels of fever induction and weight loss in ferrets were observed with both viruses (**Table 1**). This pattern of transmission suggests that a lysine at HA position 219 enhances transmission of the 2009 H1N1 virus through the air.

**Table 1:** Replication and transmission of *CA04/09* Ile219->Lys HA mutant virus in ferrets.

	Inoculated Animals				RD Contact Animals^a^			
Virus	Weight Loss (%)^c^	Virus in nasal wash^d^	Sneezing^e^	Sero-conversion^f^	Weight Loss (%)^c^	Virus in nasal wash^d^	Sneezing^e^	Sero-conversion^f^
***rgCA04/09 HA Ile219Lys*** b	13.1	3/3 (6.4)	1/3	3/3 (640–1280)	8.5	3/3 (6.9)	1/3	3/3 (640–1280)
***rgCA04/09***	8.0	3/3 (6.6)	2/3	3/3 (640–1280)	7.1	2/3 (5.5)	1/3	2/3 (80–160)

a RD, respiratory droplet.

b This experiment was performed using virus that was obtained from plaque purification and grown in MDCK cells.

c The percentage mean maximum weight loss observed during the first 10 days post-inoculation.

d Number of animals positive for infectious virus. Virus titers are expressed as mean log_10_ peak PFU/mL.

e Number of animals in which sneezing was observed during the first 10 days after inoculation (sneezing was infrequent for all infected animals tested).

f Hemagglutination inhibition (HI) assay was performed with homologous virus and turkey red blood cells. No. of positive animals and HI range indicated.

## Discussion

The emergence of the 2009 H1N1 virus created a global health concern given that it was a novel strain of influenza A resulting from reassortment of gene segments derived from avian, human and swine viruses. In the present study we demonstrate that a single Ile219→Lys amino acid change stabilizes the ionic molecular interaction network involving residues 219, 227 and 186 in the RBS of 2009 H1N1 HA that in turn substantially increases its human receptor affinity in comparison with wild-type HA. We also designed and tested another mutant form of *CA04/09* HA involving three mutations to make this interaction network hydrophobic (similar to that in *SC18* HA) and showed that this mutant (*CA04/09mut2*) HA had substantially higher affinity than wild-type HA (**Figure S3**). Our findings emphasize the importance of using a quantitative dose-dependent binding assay with recombinant HA in order to correlate biochemical glycan-binding affinity with molecular HA-glycan interactions. The increased human receptor affinity of the mutant HA correlated with efficient airborne transmission of a virus that harbored this HA mutation in ferrets compared with inefficient transmission of the wild-type *rgCA04/09* virus. These results highlight two important aspects of understanding evolution of 2009 H1N1 virus. First, the inter-amino acid interaction network in the RBS plays a key role in governing human receptor binding affinity of HA. Second, the ferret transmission results demonstrate that the quantitative human receptor-binding affinity of HA is one of the key factors that govern human host adaptation of the virus for efficient human-to-human transmission.

It has been estimated that around 59% of United States population has some immunity to the 2009 H1N1 virus either due to pre-existing immunity, through vaccination, or due to the infection with the pandemic virus [26]. Recent surveillance data has shown that genetic variants of the 2009 H1N1 virus, carrying mutations in HA, have already begun to dominate the later part of 2010 in Singapore, New Zealand and Australia [27]. Although these mutations were found to be in the antigenic sites of HA, the strains carrying these mutations were not antigenically different from *CA04/09* (no significant differences in hemagglutination inhibition titers using ferret anti-sera raised to these viruses). Previous studies using another H1N1 strain (A/Puerto Rico/8/34) have shown that in order to escape polyclonal neutralizing antibody challenge, this virus acquires mutations in its HA that increased glycan receptor-binding affinity [28]. Given that the 219 position is a part of the antigenic loop and that a single amino acid mutation in 219 increases human receptor-binding affinity, this mutation may occur as a part of antigenic drift in the currently circulating 2009 H1N1 strains. Moreover, the Ile219->Lys mutation requires a single nucleotide change, which also increases the probability of such a mutation to occur. In summary, our study provides a systematic approach to monitor the evolution of HA mutations in the currently circulating 2009 H1N1 viruses that would potentially result in strains with higher human receptor-binding affinity and human-to-human transmissibility.

## Materials and Methods

### Homology based structural modeling of *CA04/09* and *CA04/09mut1*

Using the SWISS-MODEL web-based automated homology-modeling platform (http://swissmodel.expasy.org/) the homology structural models of *CA04/09*, and *CA04/09mut1* were constructed. The template structure chosen by SWISS-MODEL was that of a recently solved crystal structure of 2009 H1N1 HA (PDB ID: 3LZG). The starting pose of the HA-human receptor complex was obtained by superimposing the modeled HA structure with the co-crystal structure of 1918 H1N1 HA with human receptor (PDB ID: 2WRG). The starting structural complex was subject to energy minimization (500 steps of steepest descent followed by 500 steps of conjugate gradient). The AMBER force-field was used to assign potentials and charges. The default version of AMBER was provided with the Discover module of InsightII molecular modeling suite (Accelrys, San Diego, CA).

### Cloning, baculovirus synthesis, expression and purification of HA

Briefly, recombinant baculoviruses with *WT* or mutant HA gene respectively, were used to infect (MOI = 1) suspension cultures of Sf9 cells (Invitrogen, Carlsbad, CA) cultured in BD Baculogold Max-XP SFM (BD Biosciences, San Jose, CA). The infection was monitored and the conditioned media was harvested 3–4 days post-infection. The soluble HA from the harvested conditioned media was purified using Nickel affinity chromatography (HisTrap HP columns, GE Healthcare, Piscataway, NJ). Eluting fractions containing HA were pooled, concentrated and buffer exchanged into 1X PBS pH 8.0 (Gibco) using 100K MWCO spin columns (Millipore, Billerica, MA). The purified protein was quantified using BCA method (Pierce).

### Binding of recombinant *CA04/09* and *CA04/09mut1* HAs to human tracheal tissue sections

Paraffinized human tracheal (US Biological) tissue sections were deparaffinized, rehydrated and incubated with 1% BSA in PBS for 30 minutes to prevent non-specific binding. HA was pre-complexed with primary antibody (mouse anti 6X His tag, Abcam) and secondary antibody (Alexa fluor 488 goat anti mouse, Invitrogen) in a molar ratio of 4∶2∶1, respectively, for 20 minutes on ice. The tissue binding was performed over different HA concentrations by diluting the pre-complexed HA in 1% BSA-PBS. Tissue sections were then incubated with the HA-antibody complexes for 3 hours at RT. The tissue sections were counterstained by propidium iodide (Invitrogen; 1∶100 in TBST). The tissue sections were mounted and then viewed under a confocal microscope (Zeiss LSM510 laser scanning confocal microscopy). In the case of sialidase pretreatment, tissue sections were incubated with 0.2 units of Sialidase A (recombinant from *Arthrobacter ureafaciens*, Prozyme) for 3 hours at 37°C prior to incubation with the proteins. This enzyme has been demonstrated to cleave the terminal Neu5Ac from both Neu5Acα2→3Gal and Neu5Acα2→6Gal motifs.

### Dose dependent direct binding of *CA04/09* and *CA04/09mut1* HAs

To investigate the multivalent HA-glycan interactions a streptavidin plate array comprising of representative biotinylated α2→3 and α2→6 sialylated glycans was used as described previously [10] (**Table S1**). 3′SLN, 3′SLN-LN, 3′SLN-LN-LN are representative avian receptors. 6′SLN and 6′SLN-LN are representative human receptors. The biotinylated glycans were obtained from the Consortium of Functional Glycomics through their resource request program. Streptavidin-coated High Binding Capacity 384-well plates (Pierce) were loaded to the full capacity of each well by incubating the well with 50 µl of 2.4 µM of biotinylated glycans overnight at 4°C. Excess glycans were removed through extensive washing with PBS. The trimeric HA unit comprises of three HA monomers (and hence three RBS, one for each monomer). The spatial arrangement of the biotinylated glycans in the wells of the streptavidin plate array favors binding to only one of the three HA monomers in the trimeric HA unit. Therefore in order to specifically enhance the multivalency in the HA-glycan interactions, the recombinant HA proteins were pre-complexed with the primary and secondary antibodies in the molar ratio of 4∶2∶1 (HA: primary: secondary). The identical arrangement of 4 trimeric HA units in the pre-complex for all the HAs permit comparison between their glycan binding affinities.

A stock solution containing appropriate amounts of Histidine tagged HA protein, primary antibody (Mouse anti 6X His tag IgG) and secondary antibody (HRP conjugated goat anti Mouse IgG (Santacruz Biotechnology) in the ratio 4∶2∶1 and incubated on ice for 20 min. Appropriate amounts of pre-complexed stock HA were diluted to 250 µl with 1% BSA in PBS. 50 µl of this pre-complexed HA was added to each of the glycan-coated wells and incubated at room temperature for 2 hrs followed by the above wash steps. The binding signal was determined based on HRP activity using Amplex Red Peroxidase Assay (Invitrogen, CA) according to the manufacturer's instructions. The experiments were done in triplicate. Minimal binding signals were observed in the negative controls including binding of pre-complexed unit to wells without glycans and binding of the antibodies alone to the wells with glycans. The binding parameters, cooperativity (*n*) and apparent binding constant (*K_d_'*), for HA-glycan binding were calculated by fitting the average binding signal value (from the triplicate analysis) and the HA concentration to the linearized form of the Hill equation:
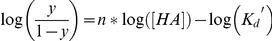
where y is the fractional saturation (average binding signal/maximum observed binding signal). The theoretical *y* values calculated using the Hill equation:
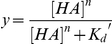
(for the set of *n* and *K_d_'* parameters) were plotted against the varying concentration of HA to obtain the binding curves for the representative human receptor (6′SLN-LN) in **Figure 2C**.

### Construction of plasmids

The eight reverse-genetics plasmids used for the rescue of recombinant influenza A/California/04/2009 (Cal/09) virus—pDZ-Cal04-PB2, pDZ-Cal04-PB1, pDZ-Cal04-PA, pDZ-Cal04-NP, pDZ-Cal04-HA, pDZ-Cal04-NA, pDZ-Cal04-M, and pDZ-Cal04-NS - were constructed by methods described previously [29], [30]. The HA plasmid pDZ-Cal04-Ile219->Lys was a derivative of the wild-type (WT) HA segment. The mutation Ile219->Lys was created by altering the position 219 codon from ATA to AAA using Stratagene QuickChange ^site^-directed mutagenesis kit and pDZ-Cal04-HA as template.

### Rescue of recombinant influenza A viruses

The influenza A Cal/04 viruses were rescued as described previously [31]. Briefly, 293T cells were transfected with the eight pDZ-Cal04 vectors encoding each of the viral genomic RNA segments. At 12 h post transfection, the 293T cells were co-cultured with MDCK cells. The rescued viruses were further isolated by plaque purification on MDCK cells. The coding sequences of each PDZ construct and viruses generated by reverse genetics were confirmed by automated sequencing performed at the Centers for Disease Control and Prevention sequencing core facility.

### Inoculation of ferrets

The 2009 H1N1 virus animal experiments were conducted under biosafety level 3 enhanced (BSL3+) laboratory conditions. Male Fitch ferrets (Triple F Farms), 6 to 12 months of age that were serologically negative by hemagglutination inhibition (HI) assay for currently circulating influenza viruses were used in this study. At the start of each experiment, all ferrets had body weights greater than 1.0 kg, with a range of 1.01–1.57 kg. Ferrets were housed throughout each experiment in cages within a Duo-Flo Bioclean mobile clean room (Lab Products, Inc. Seaford, DE). Baseline serum, temperature and weight measurements were obtained prior to infection. Temperatures were measured using a subcutaneous implantable temperature transponder (BioMedic Data Systems, Inc.). Ferrets were inoculated intranasally with 10^6^ PFU (plaque forming units) in a 1 ml volume, a dose reported to consistently infect ferrets with human or avian influenza viruses [32]. Ferrets were monitored for changes in body temperature and weight, and the presence of the following clinical signs: sneezing, lethargy, anorexia, nasal or ocular discharge, dyspnea, diarrhea, neurological dysfunction [33]. Nasal washes were collected every other day for at least 9 days post inoculation (p.i.) or contact (p.c.), analyzed [34] and titrated by standard plaque assay.

### Ethics Statement

All animal research described in this study was specifically approved by CDC's Institutional Animal care and Use Committee (IACUC). The animal research was conducted under the guidance of CDC's IACUC and in an Association for Assessment and Accreditation of Laboratory Animal Care International-accredited facility.

### Transmission experiments

For the respiratory droplet transmission experiments, ferrets were housed in adjacent transmission cages, each modified so that a side wall was replaced with a stainless-steel, perforated wall with holes 1–5 mm in diameter and spaced 3 mm apart to facilitate the transfer of respiratory droplets through the air while preventing direct contact between ferrets and indirect contact with the bedding and food of neighbouring ferrets. A total of six ferrets were used for each respiratory droplet transmission experiment [33]. Twenty-four hours after inoculation (day 1 p.i. for the inoculated ferrets and day 0 p.c. for the contact ferrets), three naive ferrets were each placed in a cage adjacent to an inoculated ferret. To prevent inadvertent physical transmission of virus by the investigators, the contact ferrets were always handled first, and all items that came into contact with the ferrets or their bedding were decontaminated between each ferret. The use of the term “respiratory droplet transmission” throughout this report refers to transmission in the absence of direct or indirect contact and does not imply an understanding of the droplet size involved in virus spread between ferrets. The ability of each virus to undergo respiratory droplet transmission among ferrets was assessed by measuring virus titres in nasal washes from contact animals every other day for at least 11 days. HI analysis was also performed on post-exposure ferret sera collected 18–21 days p.c. using 0.5% turkey erythrocytes against homologous virus [34].

## Supporting Information

Table S1 Expanded nomenclature of glycans used in the glycan array.
(DOC)

Figure S1 **ClustalW2 Sequence alignment** ***CA04/09*** **,** ***SC18*** **and** ***Bris07*** **HAs.** Only the HA1 part of the HA comprising of the RBS is shown. The hallmark Asp190 and Asp225 residues are highlighted in *green*. Residue positions highlighted in *gray* represent the residues involved in positioning Asp190. Residue positions 219 and 227 are highlighted in *yellow* to indicate the mismatched combination of residues in *CA04/09* HA.
(PDF)

Figure S2 **Dose-dependent direct glycan array binding of** ***rgCA04/09 HA Ile219Lys*** **mutant virus.** The same glycan array platform was used as that for the analysis with the HA. 50 µl of the viruses (in HAU) (diluted in 1X PBS +1% BSA) were added to each of the glycan – coated wells and incubated overnight at 4°C (to prevent sialic acid cleavage by viral neuraminidase). The bound viruses were detected with ferret anti – *CA04/09* antisera (1∶500 diluted in 1X PBS +1% BSA) and the secondary antibody (goat anti – ferret HRP conjugated antibody from Abcam; 1∶500 diluted in 1X PBS +1% BSA). The binding signals were determined based on the HRP activity of the secondary antibody, using the Amplex Red Peroxidase Assay (Invitrogen) according to the manufacturer's instructions. The assays were performed in duplicate and appropriate controls were included. The binding profile of this virus is consistent with that of the recombinant *CA04/09mut1* HA shown in **Figure 2B**.
(PDF)

Figure S3 **Design and glycan array binding of** ***CA04/09mut2*** **HA.** This mutant was designed to make the interaction network involving residues 186, 219 and 227 hydrophobic. *CA04/09mut2* HA was recombinantly expressed and analyzed on the glycan array similar to *CA04/09mut1* HA. **A**, shows stereo rendering of the RBS of *CA04/09mut2* HA interacting with the human receptor (stick representation with carbon atom colored in orange). Three mutations Ser186Pro, Ala189Thr and Glu227Ala in *CA04/09mut2* HA make the inter-amino acid interaction network identical to that observed in *SC18* HA (**Figure S1**). The mutated residues are highlighted in *red*. **B**, shows comparison of 6′SLN-LN binding curve between *CA04/09* and *CA04/09mut2* HA. Fixing the interaction network by making it hydrophobic also substantially improves human receptor binding affinity.
(PDF)
