# Supplementary material for: A Single Base-Pair Change in 2009 H1N1 Hemagglutinin Increases Human Receptor Affinity and Leads to Efficient Airborne Viral Transmission in Ferrets
Source: PLoS One. 2011 Mar 2;6(3):e17616. doi: 10.1371/journal.pone.0017616 (PMC3047569; doi:10.1371/journal.pone.0017616)
Supplement: Table S1 — Expanded nomenclature of glycans used in the glycan array. (DOC) [file pone.0017616.s001.doc]

**Table S1. Expanded nomenclature of glycans used in the glycan array**

| **Glycan** | **Expanded nomenclature** |
| --- | --- |
| 3’SLN | Neu5Acα2-3Galβ1-4GlcNAcβ1- |
| 6’SLN | Neu5Acα2-6Galβ1-4GlcNAcβ1- |
| 3’SLN-LN | Neu5Acα2-3Galβ1-4GlcNAcβ1-3Galβ1-4GlcNAcβ1- |
| 6’SLN-LN | Neu5Acα2-6Galβ1-4GlcNAcβ1-3Galβ1-4GlcNAcβ1- |
| 3’SLN-LN-LN | Neu5Acα2-3Galβ1-4GlcNAcβ1-3Galβ1-4GlcNAcβ1-3Galβ1-4GlcNAcβ1- |

Key: Neu5Ac: N-acetyl D-neuraminic acid; Gal: D-galactose; GlcNAc: N-acetyl D-glucosamine. α / β: anomeric configuration of the pyranose sugars. All the sugars are linked via a spacer to biotin (-Sp-LC-LC-Biotin as described in <http://www.functionalglycomics.org/static/consortium/resources/resourcecored5.shtml>)
