# Supplementary material for: A Single Base-Pair Change in 2009 H1N1 Hemagglutinin Increases Human Receptor Affinity and Leads to Efficient Airborne Viral Transmission in Ferrets
Source: PLoS One. 2011 Mar 2;6(3):e17616. doi: 10.1371/journal.pone.0017616 (PMC3047569; doi:10.1371/journal.pone.0017616)
Supplement: Figure S1 — ClustalW2 Sequence alignment CA04/09 , SC18 and Bris07 HAs. Only the HA1 part of the HA comprising of the RBS is shown. The hallmark Asp190 and Asp225 residues are highlighted in green. Residue positions highlighted in gray represent the residues involved in positioning Asp190. Residue positions 219 and 227 are highlighted in yellow to indicate the mismatched combination of residues in CA04/09 HA. (PDF) [file pone.0017616.s002.pdf]

Figure S1.

```
CA04/09               MKAILVVLLYTFATANADTLCIGYHANNSTDTVDTVLEKNVTVTHSVNLLEDKHNGKLCK
A/SC/1/18             MEARLLVLLCAFAATNADTICIGYHANNSTDTVDTVLEKNVTVTHSVNLLEDSHNGKLCK
A/Brisbane/59/07      MKVKLLVLLCTFTATYADTICIGYHANNSTDTVDTVLEKNVTVTHSVNLLENSHNGKLCL
                      *:. *:*** :*::: ***:*******************************:.******

CA04/09               LRGVAPLHLGKCNIAGWILGNPECESLSTASSWSYIVETPSSDNGTCYPGDFIDYEELRE
A/SC/1/18             LKGIAPLQLGKCNIAGWLLGNPECDLLLTASSWSYIVETSNSENGTCYPGDFIDYEELRE
A/Brisbane/59/07      LKGIAPLQLGNCSVAGWILGNPECELLISKESWSYIVEKPNPENGTCYPGHFADYEELRE
                      *:*:***:**:*.:***:******: * : .*******....:*******.* *******

CA04/09               QLSSVSSFERFEIFPKTSSWPNHDSNKGVTAACPHAGAKSFYKNLIWLVKKGNSYPKLSK
A/SC/1/18             QLSSVSSFEKFEIFPKTSSWPNHETTKGVTAACSYAGASSFYRNLLWLTKKGSSYPKLSK
A/Brisbane/59/07      QLSSVSSFERFEIFPKESSWPNHTVT-GVSASCSHNGESSFYRNLLWLTGKNGLYPNLSK
                      *********:****** ******  . **:*:*.: * .***:**:**. *.. **:***

                                         186  190                          219   225
CA04/09               SYINDKGKEVLVLWGIHHPSTSADQQSLYQNADTYVFVGSSRYSKKFKPEIAIRPKVRDQ
A/SC/1/18             SYVNNKGKEVLVLWGVHHPPTGTDQQSLYQNADAYVSVGSSKYNRRFTPEIAARPKVRDQ
A/Brisbane/59/07      SYANNKEKEVLVLWGVHHPPNIGDQKALYHTENAYVSVVSSHYSRKFTPEIAKRPKVRDQ
                      ** *:* ********:***..  **::**:. ::** * **:*.::*.**** *******

                      227
CA04/09               EGRMNYYWTLVEPGDKITFEATGNLVVPRYAFAMERNAGSGIIISDTPVHDCNTTCQTPK
A/SC/1/18             AGRMNYYWTLLEPGDTITFEATGNLIAPWYAFALNRGSGSGIITSDAPVHDCNTKCQTPH
A/Brisbane/59/07      EGRINYYWTLLEPGDTIIFEANGNLIAPRYAFALSRGFGSGIINSNAPMDKCDAKCQTPQ
                       **:******:****.* ***.***:.* ****:.*. ***** *:: :..*::.****:

CA04/09               GAINTSLPFQNIHPITIGKCPKYVKSTKLRLATGLRNIPSIQSR
A/SC/1/18             GAINSSLPFQNIHPVTIGECPKYVRSTKLRMATGLRNIPSIQSR
A/Brisbane/59/07      GAINSSLPFQNVHPVTIGECPKYVRSAKLRMVTGLRNIPSIQSR
                      ****:******:**:***:*****:*:***:.************
```
