# Supplementary figures and images for: A Single Base-Pair Change in 2009 H1N1 Hemagglutinin Increases Human Receptor Affinity and Leads to Efficient Airborne Viral Transmission in Ferrets
Source: PLoS One. 2011 Mar 2;6(3):e17616. doi: 10.1371/journal.pone.0017616 (PMC3047569; doi:10.1371/journal.pone.0017616)

Figure S2

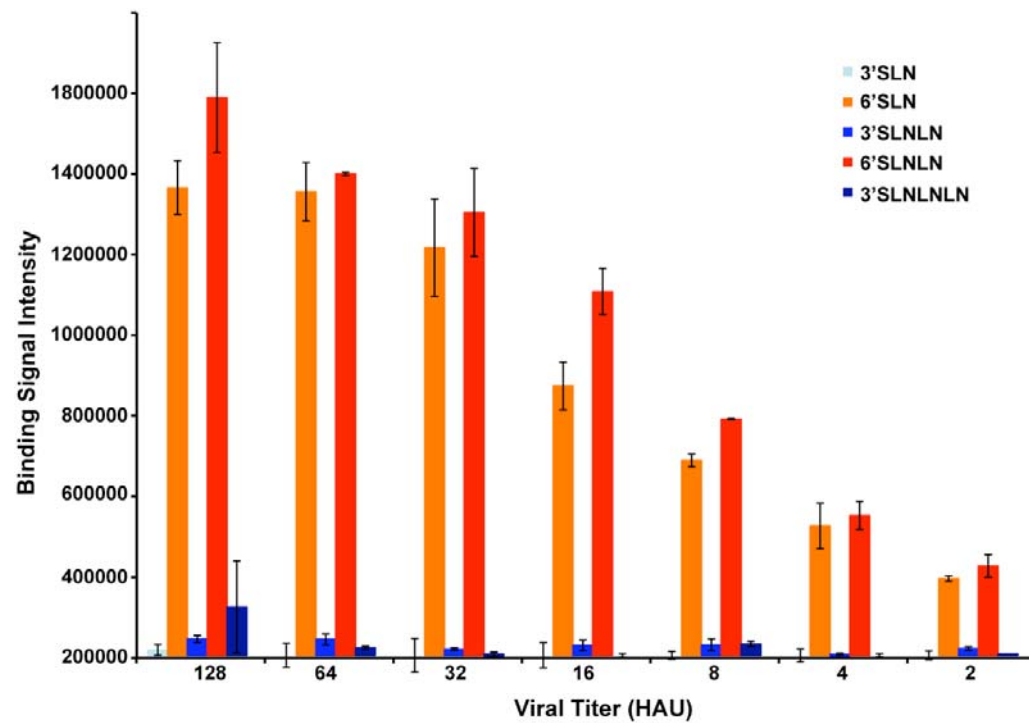

Supplement: Figure S2 — Dose-dependent direct glycan array binding of rgCA04/09 HA Ile219Lys mutant virus. The same glycan array platform was used as that for the analysis with the HA. 50 µl of the viruses (in HAU) (diluted in 1X PBS +1% BSA) were added to each of the glycan – coated wells and incubated overnight at 4°C (to prevent sialic acid cleavage by viral neuraminidase). The bound viruses were detected with ferret anti – CA04/09 antisera (1∶500 diluted in 1X PBS +1% BSA) and the secondary antibody (goat anti – ferret HRP conjugated antibody from Abcam; 1∶500 diluted in 1X PBS +1% BSA). The binding signals were determined based on the HRP activity of the secondary antibody, using the Amplex Red Peroxidase Assay (Invitrogen) according to the manufacturer's instructions. The assays were performed in duplicate and appropriate controls were included. The binding profile of this virus is consistent with that of the recombinant CA04/09mut1 HA shown in Figure 2B . (PDF) [file pone.0017616.s003.pdf]

Figure S3

A

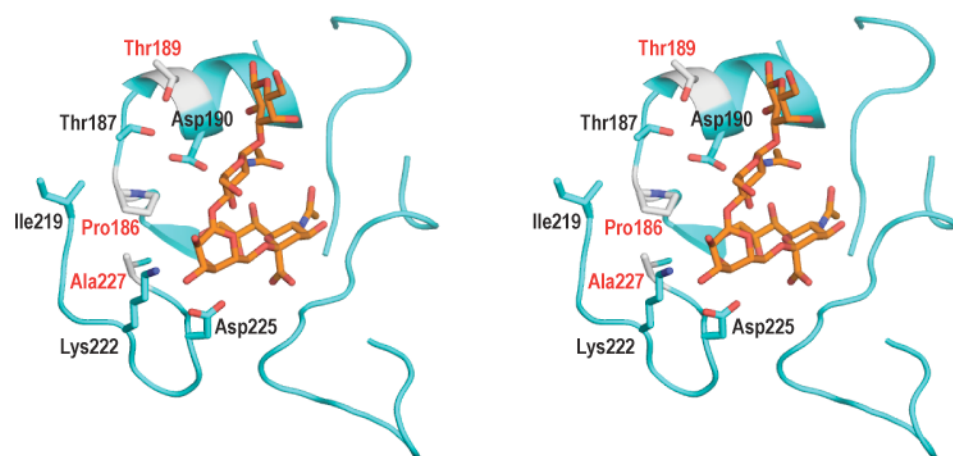

B

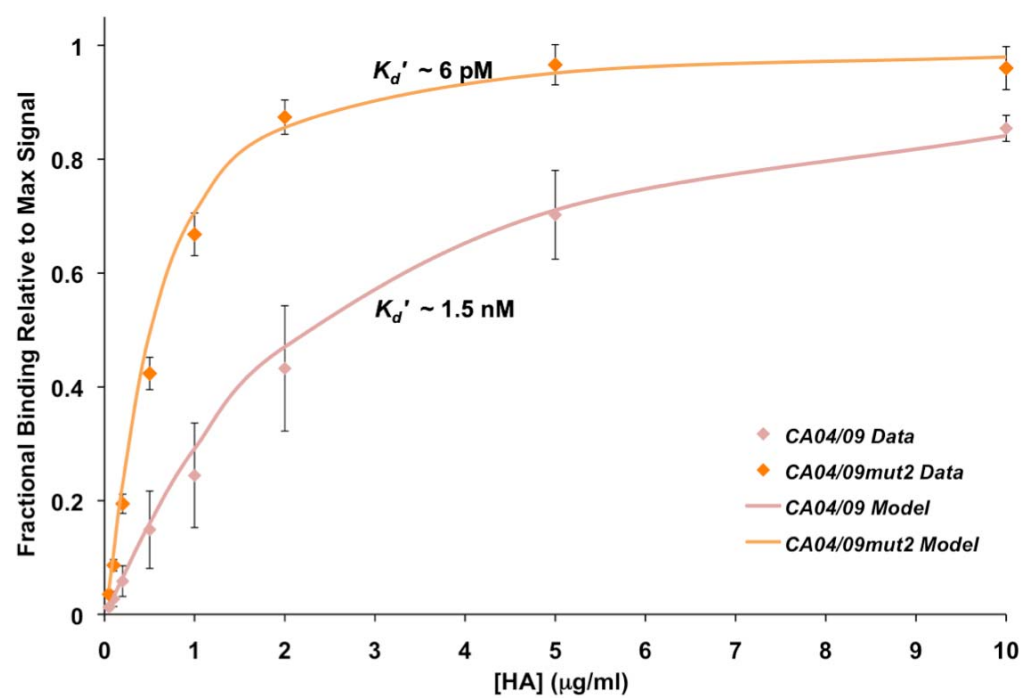

Supplement: Figure S3 — Design and glycan array binding of CA04/09mut2 HA. This mutant was designed to make the interaction network involving residues 186, 219 and 227 hydrophobic. CA04/09mut2 HA was recombinantly expressed and analyzed on the glycan array similar to CA04/09mut1 HA. A, shows stereo rendering of the RBS of CA04/09mut2 HA interacting with the human receptor (stick representation with carbon atom colored in orange). Three mutations Ser186Pro, Ala189Thr and Glu227Ala in CA04/09mut2 HA make the inter-amino acid interaction network identical to that observed in SC18 HA (Figure S1). The mutated residues are highlighted in red. B, shows comparison of 6′SLN-LN binding curve between CA04/09 and CA04/09mut2 HA. Fixing the interaction network by making it hydrophobic also substantially improves human receptor binding affinity. (PDF) [file pone.0017616.s004.pdf]
